# Study of Morphological Features and Determination of the Fatty Acid Composition of the Microalgae Lipid Complex

**DOI:** 10.3390/biom10111571

**Published:** 2020-11-19

**Authors:** Vyacheslav Dolganyuk, Anna Andreeva, Ekaterina Budenkova, Stanislav Sukhikh, Olga Babich, Svetlana Ivanova, Alexander Prosekov, Elena Ulrikh

**Affiliations:** 1 Institute of Living Systems, Immanuel Kant Baltic Federal University, A. Nevskogo Street 14, 236016 Kaliningrad, Russia; dolganuk_vf@mail.ru (V.D.); AnnaAndreeva@kantiana.ru (A.A.); abudenkova@kantiana.ru (E.B.); stas-asp@mail.ru (S.S.); olich.43@mail.ru (O.B.); 2 Department of Bionanotechnology, Kemerovo State University, Krasnaya Street 6, 650043 Kemerovo, Russia; 3 Natural Nutraceutical Biotesting Laboratory, Kemerovo State University, Krasnaya Street 6, 650043 Kemerovo, Russia; 4 Department of General Mathematics and Informatics, Kemerovo State University, Krasnaya Street 6, 650043 Kemerovo, Russia; 5 Laboratory of Biocatalysis, Kemerovo State University, Krasnaya Street 6, 650043 Kemerovo, Russia; a.prosekov@inbox.ru; 6 Kuzbass State Agricultural Academy, Markovtseva Street 5, 650056 Kemerovo, Russia; elen.ulrich@mail.ru

**Keywords:** microalgae, lipids, fatty acid composition, purification, lipid complex

## Abstract

Microalgae are rich in nutrients and biologically active substances such as proteins, carbohydrates, lipids, vitamins, pigments, phycobiliproteins, among others. The lipid composition of the microalgae *Chlorella vulgaris*, *Arthrospira platensis*, and *Dunaliella salina* was screened for the first time. The proposed method for purifying the lipid complex isolated from microalgae’s biomass involved dissolving the lipid-pigment complex in *n*-hexane for 4 h and stirring at 500 rpm. We found that the largest number of neutral lipids is contained in the biomass of microalgae *Arthrospira platensis*, fatty acids, polar lipids (glycerophospholipids), and unsaponifiable substances—in the biomass of microalgae *Dunaliella salina*, chlorophyll, and other impurities—in the biomass of microalgae *Chlorella vulgaris*. The developed method of purification of the fatty acid composition of the microalgae lipid complex confirmed the content of fatty acids in microalgae, which are of interest for practical use in the production of biologically active components. We also determined the potential of its use in the development of affordable technology for processing microalgae into valuable food and feed additives.

## 1. Introduction

Microalgae cultivation is attracting more research due to its ability to synthesize various biologically active substances, the rapid growth of biomass, and the possibility of adjusting their biochemical composition depending on the cultivation conditions [1,2,3]. Unlike heterotrophic microorganisms, which require various organic compounds for growth, single-celled photosynthetic organisms produce biomass from fully oxidized inorganic substances and mineral elements due to light energy converted during photosynthesis [4]. Furthermore, the microalgae biomass production technologies do not pollute the environment, use carbon dioxide, emit oxygen, consume a relatively small amount of water, and they can occupy land resources unsuitable for crop cultivation [1].

Currently, there are two main areas of microalgae use, that is, the production of biomass as a dietary supplement (DS), as well as the cultivation of microalgae for the subsequent isolation of biologically active substances (BAS) from the biomass [5].

Microalgae are rich in nutrients and biologically active substances such as proteins, carbohydrates, lipids, vitamins, pigments, phycobiliproteins, etc. Biologically active substances from microalgae can exhibit antioxidant, immunostimulating, antibacterial, antiviral, antitumor, antihypertensive, regenerative, and neuroprotective effects [6,7,8]. These compounds are required in medicine, cosmetology, food industry, fish farming, energy production, agriculture, feed, and functional food production [2].

Microalgae include a wide range of autotrophic organisms that grow in photosynthesis, the same as plants. The microalgae classification is revised continuously due to new genetic data. Nevertheless, two main groups of microalgae are distinguished: prokaryotic and eukaryotic [9,10].

Prokaryotic microalgae are cyanobacteria (blue-green algae). The taxonomy includes five orders: chroococcales, oscillatoriales, nostocales, stigonematales, and pleurocapsales. Prochlorales, or free-acting chloroplasts, are another group of cyanobacteria that represent an artificial group based on their different pigmentation due to the absence of phytobiliproteins and, in most cases, due to the presence of chlorophylls a and b. One of cyanobacteria’s main characteristics is their rapid absorption and accumulation of nutrients and compounds such as phosphates, cyanophycin (a polymer of aspartic acid and arginine), and branched α-1,4 polyglucan [11,12,13]. Some of these microorganisms can produce neurotoxins and hepatotoxins, while others produce therapeutic compounds, such as antiviral drugs (fucoxanthin), immunomodulators (immunostimulants; prophylactic drugs that enhance the body’s defenses against a particular infection), acetylcholinesterase inhibitors. The most important cyanobacteria used in biotechnology are *Spirulina (Arhrospira) platensis*, *Nostoc commune*, and *Aphanizomenon flosaquae* [6].

Microalgae can produce various types of lipids, that is, triacylglycerols, phospholipids, glycolipids, or phytosterols, which contain fatty acids in the range from C12 to C24 (lipids containing 12 to 24 carbon atoms), often with mono- and polyunsaturated fatty acids C16 and C18 [14]. The lipid content of microalgae varies from 20% to 50% of dry weight. These lipids can be used for energy storage, as energy substrates, as structural components of the cell membrane, and for metabolic processes (signal transduction, transcriptional and translational control, intercellular interactions, secretion, and transfer of vesicles) [15]. For example, phospholipids are found in the extrachloroplast membranes, and glycolipids are found in cell membranes; triacylglycerols can accumulate in lipid bodies in the cytoplasm and in some green algae, such as *Dunaliella*. They can also be found in the interthylakoid space of the chloroplast [16].

Some microalgae are called “oily”. The term “oily” is used to describe algae capable of accumulating large amounts of oil (>20% of dry biomass), for example, *Chlorella* sp. and *Nannochloropsis* sp., in which this indicator can reach up to 80% of dry biomass [16].

The number of lipids and the presence or position of double bonds in the carbon chain can vary depending on the microalgae species and the culture conditions. Optimal conditions can promote the conversion of fatty acids to glycerol-based membrane lipids, while unfavorable conditions can increase the synthesis of neutral lipids such as triacylglycerols. Typically, many microalgae contain polyunsaturated fatty acids (PUFAs) such as eicosapentaenoic acid, arachidonic acid, and docosahexaenoic acid. Note that palmitic acid is the predominant fatty acid in microalgae [6].

In the course of their vital activity, cyanobacteria synthesize saturated, monounsaturated, and polyunsaturated fatty acids, but the main fatty acids that accumulate in the biomass of cyanobacteria are palmitic acid and oleic acid. The microalga *Spirulina* accumulates linoleic and γ-linolenic acids in its biomass [17].

The importance of lipids derived from microalgae lies in their commercial value as an alternative food source for producing functional products from their PUFAs such as eicosapentaenoic acid (EPA), docosahexaenoic acid (DHA), as well as their precursor α-linolenic acid.

Most microalgae high in ω-3 are marine species such as *Schizochytrium* sp. and *Nannochloropsis* sp. However, freshwater species such as *Desmodesmus* sp. have also been studied as sources of omega-3 long-chain PUFAs, EPA, and DHA acids [18,19].

Depending on the cultivation conditions and the composition of the nutrient medium, the fatty acid profile can vary for the same microalgae species. For example, Scharff et al. [20] evaluated the effect of the photoperiod on the biochemical profile of the microalgae *Chlorella vulgaris* and *Scendesmus obliquus* and found that longer photoperiods (24:0, 22:2, 20:4) can reduce the synthesis of α-linolenic acid and induce the synthesis of linoleic acid, which more distinctly observed for *Chlorella vulgaris* than for *Scendedmus obliquus*. On the other hand, it was established in Reference [21] that the linoleic and α-linolenic acids’ content increases with increasing light intensity, which is a continuous illumination condition for an optimal photoperiod.

This work aims to study the qualitative and quantitative components of lipid fractions of typical microalgae found in the Kaliningrad region’s Baltic Sea and water bodies (Russia).

## 2. Materials and Methods

### 2.1. Microalgae Sampling

Samples of natural sources (water, sand, soil) were taken to take samples of microalgae. The selection of natural samples was carried out in the period from October 2019 to December 2019 in various regions of the Kaliningrad Oblast, Russia (Lake Vištytis (54°25′37″ N 22°43′30″ E), Lake Chaika (56°03′49″ N 29°04′50″ E), Lake Yantarnoye (56°01′44″ N 30°44′03″ E), Curonian Lagoon (55°07′00″ N 21°01′00″ E), Strait of Baltiysk (59°43′ N 28°24′ E), Baltic Sea coast (54°42.4′0″ N, 20°30.4′0″ E), Lake Krasnoye (54°25′59″ N, 22°30′27″ E)).

Microalgae were sampled with a box-shaped bottom sampler developed at the Institute for Biology of Inland Waters of the Russian Academy of Sciences (IBIW, Borok, Russia), covering a square area of the bottom 160 × 160 mm in size with a maximum immersion depth of 440 mm in bottom sediments; a 400 mm^2^ sample was taken. Immediately after transportation to the shore, test scores were taken using plastic tubes with an inner diameter of 45 mm. The tubes were sealed at both ends and stored in an upright position at +4 °C. The core was cut lengthwise and halved in the laboratory using two thin stainless steel plates inserted into the cut. The core halves were then divided into transverse samples (slices) with a step of 5–10 mm. All samples were stored at −20 °C in the dark, in plastic bags with squeezed air, from which samples of microalgae were taken for research.

Further, the isolation of pure microalgae cultures and identification of microalgae strains capable of actively accumulating biomass and target products (lipids, proteins, and carbohydrate-mineral complex), as well as those suitable for cultivation in laboratory conditions, were carried out.

The pure microalgae cultures were isolated from enrichment cultures in which their growth was observed. The studied samples of natural sources (water, sand, soil) were introduced into a standard BBM nutrient medium (Stylab, Moscow, Russia) to obtain enrichment cultures. Within this research framework, 128 samples of natural sources, taken in various regions of the Kaliningrad region, were used, of which 27 samples showed the growth and development of microalgae at the initial stage of obtaining enrichment cultures.

Partial sequences of the 18S and/or 16S rRNA gene (Appendix A, Appendix B and Appendix C) were determined to identify isolated from the enrichment culture strains of microorganisms (microalgae), after which a comparative analysis was performed with the known sequences from the GenBank database. The results of a comparative analysis of the 18S rRNA gene sequence indicated that the following microalgae were isolated from natural sources (soil, water, sand): *Chlorella vulgaris*; *Arthrospira platensis*; *Dunaliella salina.*

### 2.2. Microalgae Biomass Cultivation

The microalgae cultivation process and biomass production were carried out at room temperature (21–23 °C) and constant illumination of 30–50 μE with fluorescent lamps with warm white light for 7 days.

Tamiya medium was used for the cultivation of *Chlorella vulgaris*; Zarruk nutrient medium was used for cultivation and production of biomass of microalgae *Arthrospira platensis*; Omarov’s medium was used for the production of biomass of the microalgae *Dunaliella salina*. The culture media were sterilized by autoclaving, the microelements of the Zarruk medium were sterilized by filtration through a filter with a pore diameter of 0.22 μm and added after autoclaving into culture media cooled to room temperature. Compositions of nutrient media used for cultivation and production of microalgae biomass are presented in Table 1. The cultivation of microalgae was carried out until the studied samples’ required amount of biomass was obtained.

### 2.3. Determination of the Microalgae Morphology

The morphology of microalgae was determined at 40× magnification using a binocular microscope MC-300 (Micros, Vienna, Austria).

### 2.4. Isolation of the Microalgae Lipid Complex

Isolation of the lipid complex was carried out as follows. At the initial stage, the biomass of cell microcultures was subjected to centrifugation. The process was carried out for 20–25 min with an acceleration of 2000 rpm. The resulting precipitate was then dried in a drying chamber for 24 ± 1 h at a drying temperature of 55 ± 2 °C. After that, the cell walls were destroyed using an Elmasonic S30 (Elma, Singen, Germany) ultrasonic cleaning unit with cover and basket for 0.5 min. The resulting biomass underwent the extraction of the lipid-pigment complex. The complex was extracted on an R 104 S-SK Soxhlet extractor (Behr Labor-Technik, Düsseldorf, Germany) in the presence of the ethanol and petroleum ether mixture as an extractant in a 2:1 ratio. The extraction process was carried out for 8 h. Then, the resulting mixture was subjected to vacuum evaporation in a Rotavapor R-300 rotary evaporator (Buchi Labortechnik AG, Flawil, Switzerland).

### 2.5. Purification of the Microalgae Lipid Complex

The purification method of the lipid complex isolated from the biomass was based on the dissolution of the complex in *n*-hexane. For this purpose, an organic solvent, *n*-hexane, was added to the obtained lipid complex after evaporation in a rotary evaporator, and then the complex was dissolved entirely (with stirring). The sample was then filtered and evaporated under vacuum in a Rotavapor R-300 rotary evaporator (Buchi Labortechnik AG, Flawil, Switzerland). During the study, the duration of the lipid-pigment complex dissolution process and the intensity of stirring were varied.

#### 2.5.1. Transparency of the Lipid Complex

Solutions of formazin, hydrazine sulfate, and urotropine were prepared for analysis. A formazin turbidity unit (FTU) was defined as an aqueous suspension of formazin diluted in a ratio of 1:1000, obtained by the interaction of equal volumes of an aqueous solution of hydrazine sulfate with a mass concentration of 10 g/L and an aqueous solution of urotropine with a mass concentration of 100 g/L. The mixture was stored for 24 h at a temperature of (20 ± 2) °C to obtain a stable suspension. The resulting suspension was used to prepare the formazin suspensions with a transparency of 2 and 50 FTU. The degree of the lipid complex transparency was determined according to the optical density and the calibration graph. Measurements for a calibration graph plotting were carried out with a PE-5400UF photocolorimeter (EkrosKhim, St. Petersburg, Russia) at wavelengths of 570 nm or 590 nm. Distilled water was poured into one cuvette of the photocolorimeter, and calibration suspensions with a transparency of 2 and 50 FTU were poured into the other two. The microalgae lipid complex was placed (without the formation of air bubbles) in the cuvette of the photocolorimeter (length 20 mm); the cuvette was quickly placed into the instrument, and the optical density was measured relative to the cuvette with the same lipid complex, but filtered through a folded filter at a temperature of 20 ± 2 °C.

#### 2.5.2. Acid Value of the Lipid Complex

An alcohol-ether mixture was prepared from two parts of diethyl ether and one part of ethanol with 5 drops of phenolphthalein solution per 50 mL of the mixture. The mixture was neutralized with a 0.1 N solution of potassium or sodium hydroxide until a barely noticeable pink color. Then, 3–5 g of the lipid complex was weighed into a conical flask, 50 mL of a neutralized solvent mixture was poured in, shaken, and, with constant shaking, rapidly titrated with 0.1 M NaOH solution of potassium or sodium hydroxide until a slightly pink color was obtained, stable for 30 s. The acid number was calculated based on the titration results.

#### 2.5.3. Mass Fraction of Non-Fatty Impurities

About 100× *g* of the microalgae lipid complex sample was dissolved in an equal amount of the solvent (*n*-hexane) and filtered through a filter. The filter was placed in a pre-prepared bag and treated with solvent in a Soxhlet apparatus until the fat was completely removed. The end of the extraction was determined by placing one drop of the solution into a watch glass (after evaporation of the ether, no greasy stain should remain on the glass).

At the end of the extraction, the bag was opened, the filter was worn down under an exhaust device to completely remove the odor of the solvent, and placed in a weighing glass. An open glass with a washed filter was placed in an oven and dried for 1 h at a temperature of 103 ± 3 °C, after which the glass was closed with a lid, cooled in a desiccator for 40 min, and weighed every 30 min of drying to constant weight. The mass fraction X (%) of non-fatty impurities was calculated by the formula:

(1) $$X=\frac{\left(m_{2}-m_{1}\right)}{m}\times 100\%$$

where *m* is the mass of the analyzed sample of the lipid complex, g; *m*_1_ is the mass of the glass with a clean filter, g; *m*_2_—the mass of the glass with filter and non-fatty impurities, g. The arithmetic mean of the results of two parallel determinations was taken as the final analysis result.

### 2.6. Determination of the Fatty Acid Composition of Microalgae

A reagent for transesterification of fatty acid triglycerides was prepared in advance. For this, 1.15 g of metallic sodium was dissolved in 25 mL of methanol, the solution was cooled, and used freshly prepared.

Then, 1.9 mL of *n*-hexane was added to a 2 mL polypropylene test tube, and 10 μL of the lipid fraction was added to the solvent. For complete transfer, the pipette tip was dipped in *n*-hexane and pipetted. For transesterification, 100 μL of sodium methoxide solution was added. Then it was stirred vigorously for 1 min on a vortex mixer. The reaction mixture was then set aside for 10 min and filtered through a 0.40 syringe filter into a vial for subsequent chromatography.

The fatty acid composition was determined in two ways: HPLC with ultraviolet detection and HPLC with mass spectrometric detection.

High-performance liquid chromatography (HPLC) with ultraviolet detection was performed on an LC-20 chromatograph (Shimadzu, Kyoto, Japan) using water and acetonitrile with added trifluoroacetic acid as a mobile phase. Detection was carried out using a diode-array detector in the detection range of 180 nm–900 nm. The flow rate of the eluent in all cases was 1 mL/min; elution was carried out in a gradient mode, the time and gradient were selected individually for each case, a mixture of prepared water (purification grade MQ) and acetonitrile with the addition of 0.1% trifluoroacetic acid was used as solvents. A Shim-pack HR-ODS column with a packing material (sorbent) particle size of 3 µm, closed on both sides by filters with a pore diameter of 2–5 µm, was used. The theoretical number of plates is approximately 30,000, the pressure tolerance is 50 MPa, the length is 200 mm, and the inner diameter is 10 mm. The injection volume was 10 μL, the columns were thermostated at temperatures up to 80–100 °C.

HPLC with mass spectrometric detection was performed additionally because this method outperforms classical HPLC in the analysis of fatty acid molecules and enables accurate quantitative analysis of fatty acid molecules. HPLC mass spectrometry allowed for the simultaneous analysis of a mixture of many fatty acids and showed its unique specificity and high speed of analysis. The HPLC chromatography with mass spectrometric detection is equipped with a chamber for the fragmentation of ionized molecules. Detection was carried out in the TIC mode over a mass spectrum of 50–800 *m*/*z*. The thermostat was heated in a gradient mode according to the following program: 0 min—80 °C; 10 min—150 °C; 30 min—250 °C; injection volume 1 μL.

### 2.7. Statistical Analysis

Each experiment was repeated three times, and data were expressed as means ± standard deviation. Data processing was carried out using standard methods of mathematical statistics. The homogeneity of the studied samples was checked using the Student’s *t*-test. The obtained data were subjected to analysis of variance (ANOVA) using the Statistica 10.0 software (StatSoft Inc., 2007, Tulsa, OK, USA). Differences between the means were considered significant when the confidence interval did not exceed 5% (*p* < 0.05).

## 3. Results

Analysis of the sequences of the 16S rRNA and 18S rRNA genes (Appendix A, Appendix B and Appendix C) made it possible to conclude that they fully corresponded to the genotypes of the microalgae *Chlorella vulgaris*, *Arthrospira platensis*, and *Dunaliella salina*. Since they are typical microalgae of the Baltic Sea and water bodies of the Kaliningrad region (Russia), their lipid potential was studied as a possible raw material basis for feed and dietary supplements [3].

### 3.1. Results of the Study of the Microalgae Morphology

**Figure 1:**
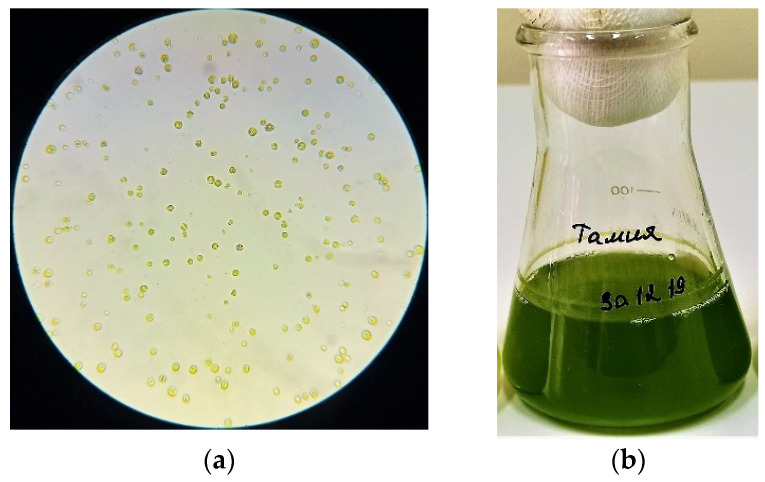
The results of studying the morphological features of the microalgae *Chlorella vulgaris* and their suspensions: (**a**) Cells under a microscope; (**b**) The suspension’s appearance.

**Figure 2:**
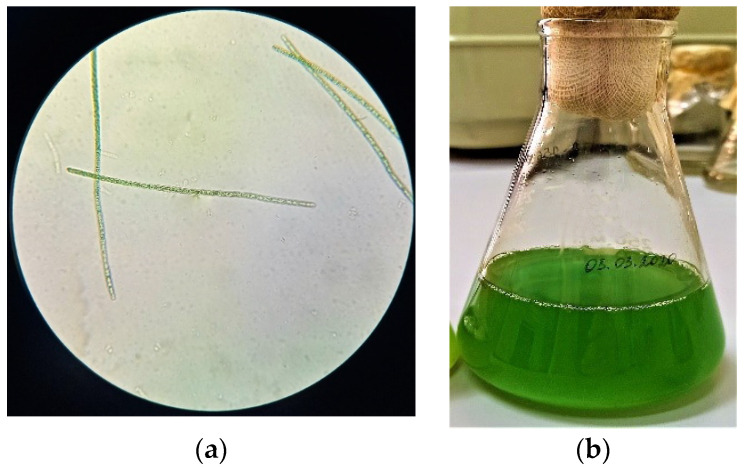
The results of studying the morphological features of the microalgae *Arthrospira platensis* and their suspensions: (**a**) Cells under a microscope; (**b**) The suspension’s appearance.

**Figure 3:**
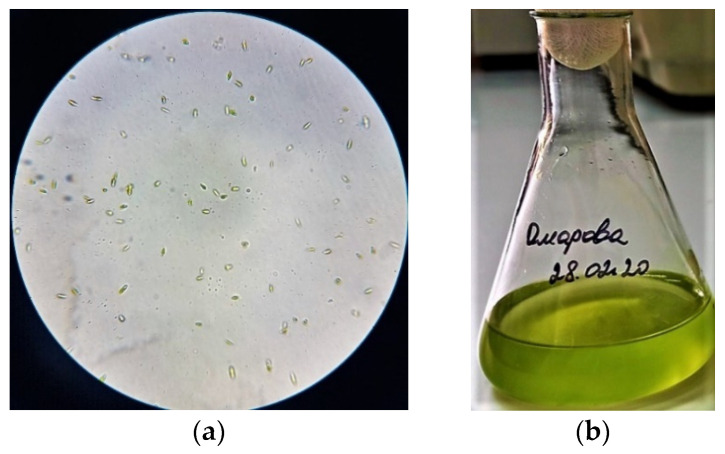
The results of studying the morphological features of the microalgae *Dunaliella salina* and their suspensions: (**a**) Cells under a microscope; (**b**) The suspension’s appearance.

The study of morphological features (Figure 1, Figure 2 and Figure 3) of isolated microalgae and their suspensions is of interest for revealing the characteristics of the biomass accumulation degree and determining the cultivation potential.

The culture of microalgae (Figure 1) is represented by cells of weakly ellipsoidal shape, ranging in size from 1.5 to 2.0 μm, green or dark green. The cell culture did not contain flagella. The chloroplast was cup-shaped. According to the analysis of morphological features obtained as a result of microalgae *Arthrospira platensis* microscopy (Figure 2), it can be concluded that the cells are ranging in size from 8.0 to 10.0 μm in length, the width of the cell varies from 2.0 to 4.5 μm. Also, it was found that the cells were poorly coiled trichomes of green or dark green color. The cells of the microalgae *Dunaliella salina* (Figure 3) have a wide-oval shape with a narrowed end and a widened posterior end. Cell size varies from 7.5 to 10.5 µm. The cells are green and have flagella.

### 3.2. Results of Purification of the Microalgae Lipid Complex

To assess the efficiency of purification of the lipid complex isolated from the biomass of microalgae (*Chlorella vulgaris*, *Arthrospira platensis*, *Dunaliella salina*), the organoleptic and physicochemical characteristics were selected, that is, the presence of precipitate, the transparency of the complex, the mass fraction of non-fatty impurities, and the acid value.

The results of the purification of the lipid complex isolated from the biomass of the microscopic algae cell cultures are presented in Table 2.

### 3.3. Results of Determining the Fatty Acid Composition of the Microalgae Lipid Fraction

The fatty acid composition of algal strains is influenced by various factors (nutrient levels, temperature, light intensity, etc.). This makes it difficult to determine a single composition profile for practical use in creating biologically active food additives and valuable feed additives for animal husbandry based on algae. In addition, clear differences in carbon chain length and degree of unsaturation are important characteristics of microalgal lipids and can influence their properties and characteristics. The qualitative and quantitative content of the lipid fraction of the lipid complex of microalgae is presented in Table 3, Table 4 and Table 5.

The maximum concentration of saturated fatty acids C16:0 was found in the microalgae *Chlorella vulgaris* and *Dunaliella salina*. In microalgae *Dunaliella salina* and *Arthrospira platensis*, the maximum concentration of unsaturated fatty acids (C16:1n-7) ranged from 14.73 to 23.31%. The lipid complex of microalgae contains neutral lipids, fatty acids, polar lipids, unsaponifiables, chlorophyllides, and other impurities. The largest number of neutral lipids and triglycerides was contained in the biomass of microalgae *Arthrospira platensis* (58.2 ± 0.8%), fatty acids, polar lipids (glycerophospholipids), and unsaponifiable substances—in the biomass of microalgae *Dunaliella salina* (30.9 ± 0.2%, 16.4 ± 0.4%, and 18.9 ± 0.5%, respectively), chlorophyll, and other impurities—in the biomass of microalgae *Chlorella vulgaris* (5.2 ± 0.1% and 55.7 ± 0.7%, respectively).

## 4. Discussion

In the course of our work, the morphological features of the microalgae *Chlorella vulgaris*, *Arthrospira platensis*, *Dunaliella salina* were determined, which are similar to a previous study [7]. In contrast to the results of Reference [9], in our samples, all cells of the microalga *Arthrospira platensis* were green, weakly folded trichomes. A prior study [17] showed that the cells of the microalga *Dunaliella salina* had sizes within a given shape and a gray-green color.

The isolated samples of microalgae are widespread in water resources and coastal areas, including in the Baltic Sea, Kaliningrad region (Russia). These samples’ biomass accumulation degree reveals their potential as raw materials for obtaining various biologically active components, including lipids, for food and feed additives.

The technological parameters of the lipid complex purification process affect the quantitative indicators and the qualitative characteristics of the product. The interdependence of the following factors (Table 2) was studied—the duration of the dissolution process of the lipid complex in *n*-hexane, the stirring speed, the type of microalgae, and the indicators of the purification degree of the lipid complex (no sediment, mass fraction of non-fatty impurities, acid number). Because empirical data on the characteristics of the degree of purification of the lipid complex did not agree with the normal distribution hypothesis, the nonparametric Kruskal-Wallis test, and the median test for independent samples was used. With probabilities ranging from 0.0001 to 0.0007 (*p* < 0.05), the hypothesis of equality of means was not confirmed for the stirring rate. Therefore, there is a statistically significant relationship between the rate of stirring the solution with the lipid complex and the degree of purification. The homogeneity check of the obtained samples of values by the t-criterion (Student’s test) showed that there are statistically significant differences between the average indicators of the characteristics of the degree of assessment at a stirring speed of 250 rpm and 500 rpm, 250 rpm and 750 rpm, but absent at stirring at 500 and 750 rpm. No statistically significant dependence (*p* > 0.05) on the process’s duration and the type of microalgae was found. For this reason, there is no need to carry out the dissolution process for 6 h.

The recommended values of the technological parameters of the purification process of the isolated microalgae’s lipid complex are the duration of the dissolution process in *n*-hexane of 4 h and the stirring speed of 500 rpm. With these technological parameters of the lipid complex purification, no precipitate and no non-fatty impurities are observed, in contrast to the method proposed in Reference [14]. This paper investigates the lipid complex’s purification for 6 h at a stirring speed of 400 rpm. A large amount of protein and pigment impurities was found when using these process parameters. The method proposed in Reference [14] is more destructive for the lipid complex of microalgae, which does not contribute to a significant yield of fatty acids. The results from determining the acid number of the lipid complex of all types of microalgae presented in the paper coincide with the results obtained in our studies.

The paper [20] indicates that the lipid complex of microalgae contains only triglycerides (49 ± 0.7%) and fatty acids in small amounts (16.9 ± 0.6%). Our results indicate (Table 4) that we used a more efficient way of isolating and purifying the lipid complex in our research.

## 5. Conclusions

The lipid composition of the microalgae *Chlorella vulgaris*, *Arthrospira platensis*, and *Dunaliella salina* was screened for the first time. The developed high-tech, affordable, and effective purification method of the fatty acid composition of the microalgae lipid complex confirmed the content of fatty acids in these microalgae, which are of interest for practical use in the creation of biologically active food supplements and valuable feed additives for livestock. The lipid fraction of the lipid complex of the microalgae *Chlorella vulgaris*, *Dunaliella salina*, and *Arthrospira platensis* contains important fatty acids valuable for the production of feed additives for animal husbandry (myristic, palmitic, oleic, stearic, and linoleic acids). The simplicity, speed, and efficiency of the developed purification method will allow, after additional research, its use in the processes of concentration, isolation, and disinfection of lipids from lyophilized biomass of microalgae with high yields and extraction purity.

These studies established that microalgal biomass is a promising product as it is a new source of lipids. The idea of using these lipids for the production of dietary supplements for humans and feed additives for animals is promising. However, it is necessary to develop technologies for producing lipids from microalgae, which would ensure their competitive cost. In this regard, the prospect of reducing the cost of cultivating microalgae and isolating lipids from them has not lost its relevance and is being actively addressed.

## Figures and Tables

**Table 1:** Composition of nutrient medium for microalgae.

Component	I	II	III	
NaCl, g/L	-	117.00	1.0	
NaHCO_3_, g/L	-	0.042	16.8	
Potassium Nitrate (KNO_3_), g/L	5.00	0.25	-	
Magnesium Sulfate Heptahydrate (MgSO_4_·7H_2_O), g/L	2.50	0.60	0.2	
NaNO_3_, g/L	-	-	2.5	
Potassium Dihydrogen Phosphate (KH_2_PO_4_), g/L	1.25	0.07	-	
K_2_HPO_4_·3H_2_O, g/L	-	-	1.0	
K_2_SO_4_, g/L	-	-	1.0	
CaCl_2_·2H_2_O, g/L	-	-	0.04	
CaCl_2_, g/L	-	0.01	-	
Fe+EDTA (30.2%) solution, mL	1.00			
Solution of trace elements of the following composition, mL	1.00			
NH_4_VO, g/L	0.023	-	-	0.023
H_3_BO_3_, g/L	2.860	2.860	2.860	-
MnCl_2_·4H_2_O, g/L	1.810	1.810	1.810	-
ZnSO_4_·7H_2_O, g/L	0.222	0.222	0.222	-
K_2_Cr_2_(SO_4_)_4_·24H_2_O, g/L	-	-	-	0.096
CuSO_4_·5H_2_O, g/L	-	-	0.080	-
NiSO_4_·7H_2_O, g/L	-	-	-	0.048
Co(NO_3_)_2_·6H_2_O, g/L	-	-	-	0.044
Ti_2_(SO_4_)_3,_ g/L	-	-	-	0.040
NH_4_VO, g/L	-	0.023	-	-
MoO_3_, g/L	0.018	0.018	0.015	-
Na_2_WO_4_·2H_2_O, g/L	-	-	-	0.018
Distilled water	up to 1.0 L			

I—Chlorella vulgaris; II—Dunaliella salina; III—Arthrospira platensis.

**Table 2:** The efficiency of the ultrafiltration of a lipid complex isolated from the microalgae biomass.

Culture	Indicators	Process Duration, h					
		4			6		
		Stirring Speed, rpm					
		250	500	750	250	500	750
I	A	+	−	−	−	−	−
	B	0.6	0.0	0.0	0.4	0.0	0.0
	C	0.6	0.2	0.2	0.5	0.2	0.2
II	A	+	−	−	−	−	−
	B	0.4	0.0	0.0	0.3	0.0	0.0
	C	0.7	0.2	0.1	0.5	0.2	0.1
III	A	+	−	−	−	−	−
	B	0.5	0.0	0.0	0.5	0.0	0.0
	C	0.8	0.2	0.1	0.6	0.2	0.1

I—*Chlorella vulgaris*; II—*Dunaliella salina*; III—*Arthrospira platensis.* A—Precipitate; B—Mass fraction of non-fatty impurities, %; C—Acid value, mg KOH. Note: “+”—precipitate is present; “−”—no precipitate.

**Table 3:** Fatty acid composition of microalgae biomass.

Fatty Acids	Percentage of Fatty Acids to the Total Dry Weight of Biomass		
	I	II	III
Saturated	34.62 ± 0.81	34.67 ± 0.56	41.92 ± 0.45
C8:0	-	3.60 ± 1.26	-
C10:0	-	2.43 ± 0.81	-
C12:0	0.70 ± 1.01	6.87 ± 0.34	-
C14:0	6.30 ± 1.12	2.77 ± 0.12	2.28 ± 0.12
C15:0	-	4.44 ± 0.45	-
C16:0	22.80 ± 0.30	9.82 ± 1.36	37.97 ± 1.95
C17:0	-	-	0.10 ± 0.15
C18:0	2.18 ± 0.20	3.07 ± 0.09	1.57 ± 0.07
C20:0	2.64 ± 0.30	1.67 ± 0.12	-
unsaturated	65.38 ± 0.69	65.08 ± 0.22	57.8 ± 0.72
C14:1	-	-	0.72 ± 0.00
C16:1n-7	23.31 ± 0.3	14.73 ± 0.21	5.43 ± 0.86
C16:2n-4	1.95 ± 0.40	-	0.37 ± 0.09
C16:3n-4	-	-	0.18 ± 0.05
C16:4n-1	-	-	0.06 ± 0.09
C18:1n-9	15.42 ± 1.80	8.01 ± 0.45	3.21 ± 0.35
C18:1n-7			2.47 ± 0.17
C18:2n-6	14.9 ± 0.14	4.80 ± 0.04	20.94 ± 0.93
C18:3n-3	1.60 ± 0.31	12.80 ± 0.36	0.05 ± 0.08
C18:3n-6	-	-	21.50 ± 1.65
C18:4n-3	-	-	0.08 ± 0.11
C20:1n-11+n-9	-	-	0.57 ± 0.52
C20:2n-6	-	-	0.17 ± 0.00
C20:3n-6	-	-	0.29 ± 0.03
C20:4n-6	2.00 ± 0.54	-	-
C20:5n-3	6.20 ± 1.34		0.48 ± 0.10
C22:1	-	9.44 ± 0.24	-
C22:1n-11	-	-	0.37 ± 0.29
C22:5n-3	-	-	0.51 ± 0.09
C22:6n-3	-	15.30 ± 0.06	0.40 ± 0.56
Total, %	100.00	99.75	99.72

I—*Chlorella vulgaris*; II—*Dunaliella salina*; III—*Arthrospira platensis.* C8:0—Caprylic acid; C10:0—Capric acid; C12:0—Lauric acid; C14:0—Myristic acid; C14:1—Myristoleic acid; C15:0—Pentadecylic acid; C16:0—Palmitic acid; C16:1—Palmitovaccenic acid; C16:2—Hexadecadienoic acid; C16:3—Hexadecatrienoic acid; C16:4—Palmitidonic acid; C18:0—Stearic acid; C18:1—Oleic acid; C18:2—Linoleic acid; C18:3—Linolenic acid; C18:4—Stearidonic acid; C20:0—Arachidic acid; C20:1—Paullinic + Gadoleic acid; C20:2—Dihomo-linoleic acid; C20:3—Dihomo-γ-linolenic acid; C20:4—Arachidonic acid; C20:5—Eicosapentaenoic acid; C22:0—Behenic acid; C22:1—Erucic acid; C22:5—Eicosapentaenoic acid; C22:6—Docosahexaenoic acid.

**Table 4:** The qualitative and quantitative content of the lipid fraction of the microalgae lipid complex.

Lipids	Microalgae		
	I	II	III
Neutral lipids	36.7 ± 0.6 ^a^	57.3 ± 0.8	58.2 ± 0.8
Triacylglycerides	14.2 ± 0.5	26.4 ± 0.6	51.1 ± 0.4
Fatty acids	22.5 ± 0.1	30.9 ± 0.2	7.1 ± 0.4
Polar lipids (glycerophospholipids) ^b^.	0.8 ± 0.1	16.4 ± 0.4	0.10 ± 0.3
Unsaponifiable substances	13.1 ± 0.1	18.9 ± 0.5	15.8 ± 0.2
Chlorophyllides ^c^.	5.1 ± 0.1	4.9 ± 0.1	14.3 ± 0.1
Other impurities ^d^.	55.7 ± 0.7	2.5 ± 0.1	4.5 ± 0.1

I—Chlorella vulgaris; II—Dunaliella salina; III—Arthrospira platensis. ^a^ Values represent triplicate standard deviations for triacylglycerides and fatty acids and bi-repeat differences for unsaponifiables and polar lipids. ^b^ Polar lipids in this table are glycolipids and phospholipids quantified by HPLC with the mass fraction of chlorophyll phytol side chains included in the unsaponifiables. ^c^ Chlorophyllide (non-phytol fragment of chlorophylls) is calculated based on the assumption that all chlorophyll pigments had the same molecular structure as chlorophyll a. ^d^ “Other impurities” is the difference between 100% and known ingredients.

**Table 5:** Fatty acid composition of the lipid fraction of the microalgae lipid complex.

Lipid Fractions	Content, mg/g		
	I	II	III
Myristic acid	0.18 ± 0.1	0.51 ± 0.1	0.14 ± 0.1
Palmitic acid	3.87 ± 0.1	2.61 ± 0.1	4.57 ± 0.1
Oleic acid	4.79 ± 0.1	3.39 ± 0.1	3.60 ± 0.1
Stearic acid	9.61 ± 0.1	5.31 ± 0.1	9.86 ± 0.1
Linoleic acid	1.62 ± 0.1	0.95 ± 0.1	1.78 ± 0.1

I—Chlorella vulgaris; II—Dunaliella salina; III—Arthrospira platensis.

## Appendix A. Sequences of the 18S Ribosomal RNA Gene of the Studied Microorganism (Sample I)

TGTAGTCATATGCTTGTCTCAAAGATTAAGCCATGCATGTCTAAGTATAAACTGCTTTATACTGTGAAACTGCGAATGGCTCATTAAATCAGTTATAGTTTATTTGATGGTACCTACTACTCGGATACCCGTAGTAAATCTAGAGCTAATACGTGCGTAAATCCCGACTTCTGGAAGGGACGTATTTATTAGATAAAAGGCCGACCGGGCTCTGCCCGACTCGCGGTGAATCATGATAACTTCACGAATCGCATGGCCTTGTGCCGGCGATGTTTCATTCAAATTTCTGCCCTATCAACTTTCGATGGTAGGATAGAGGCCTACCATGGTGGTAACGGGTGACGGAGGATTAGGGTTCGATTCCGGAGAGGGAGCCTGAGAAACGGCTACCACATCCAAGGAAGGCAGCAGGCGCGCAAATTACCCAATCCTGACACAGGGAGGTAGTGACAATAAATAACAATACTGGGCCTTTTCAGGTCTGGTAATTGGAATGAGTACAATCTAAACCCCTTAACGAGGATCAATTGGAGGGCAAGTCTGGTGCCAGCAGCCGCGGTAATTCCAGCTCCAATAGCGTATATTTAAGTTGCTGCAGTTAAAAAGCTCGTAGTTGGATTTCGGGTGGGGCCTGCCGGTCCGCCGTTTCGGTGTGCACTGGCAGGGCCCACCTTGTTGCCGGGGACGGGCTCCTGGGCTTCACTGTCCGGGACTCGGAGTCGGCGCTGTTACTTTGAGTAAATTAGAGTGTTCAAAGCAGGCCTACGCTCTGAATACATTAGCATGGAATAACACGATAGGACTCTGGCCTATCCTGTTGGTCTGTAGGACCGGAGTAATGATTAAGAGGGACAGTCGGGGGCATTCGTATTTCATTGTCAGAGGTGAAATTCTTGGATTTATGAAAGACGAACTACTGCGAAAGCATTTGCCAAGGATGTTTTCATTAATCAAGAACGAAAGTTGGGGGCTCGAAGACGATTAGATACCGTCCTAGTCTCAACCATAAACGATGCCGACTAGGGATCGGCGGATGTTTCTTCGATGACTCCGCCGGCACCTTATGAGAAATCAAAGTTTTTGGGTTCCGGGGGGAGTATGGTCGCAAGGCTGAAACTTAAAGGAATTGACGGAAGGGCACCACCAGGCGTGGAGCCTGCGGCTTAATTTGACTCAACACGGGAAAACTTACCAGGTCCAGACATAGTGAGGATTGACAGATTGAGAGCTCTTTCTTGATTCTATGGGTGGTGGTGCATGGCCGTTCTTAGTTGGTGGGTTGCCTTGTCAGGTTGATTCCGGTAACGAACGAGACCTCAGCCTGCTAAATAGTCACGGTTGGCTCGCCAGCCGGCGGACTTCTTAGAGGGACTATTGGCGACTAGCCAATGAAGCATGAGGCAATAACAGGTCTGTGATGCCCTTAGATGTTCTGGGCCGCACGCGCGCTACACTGATGCATTCAACGAGCTTAGCCTTGGCCGAGAGGCCCGGGTAATCTTTGAAACTGCATCGTGATGGGGATAGATTATTGCAATTATTAATCTTCAACGAGGAATGCCTAGTAAGCGCAAGTCATCAGCTTGCGTTGATTACGTCCCTGCCCTTTGTACACACCGCCCGTCGCTCCTACCGATTGGGTGTGCTGGTGAAGTGTTCGGATTGGCGACCGGGGGCGGTCTCCGCTCTCGGCCGCCGAGAAGTTCATTAAACCCTCCCACCTAGAGGAAGGAGAAGTCGTAACAAGGTTTCCGTAGGTGAACCTGCGGAAGGATCA

## Appendix B. Sequences of the 18S Ribosomal RNA Gene of the Studied Microorganism (Sample II)

AGAGTTTGATCCTGGCTCAGGATGAACGCTGGCGGTCTGCTTAACACATGCAAGTCGAACGGGCTCTTCGGAGCTAGTGGCGGACGGGTGAGTAACACGTGAGAATCTGGCTCCCGGTCGGGGACAACAGAGGGAAACTTCTGCTAATCCCGGATGAGCCGAAAGGTAAAAGATTTATCGCCGGGAGATGAGCTCGCGTCTGATTAGCTAGTTGGTGAGGTAAAGGCTCACCAAGGCGACGATCAGTAGCTGGTCTGAGAGGATGATCAGCCACACTGGGACTGAGACACGGCCCAGACTCCTACGGGAGGCAGCAGTGGAGAATTTTCCGCAATGGGCGCAAGCCTGACGGAGCAAGACCGCGTGGGGGAGGAAGGCTCTTGGGTTGTAAACCCCTTTTCTCAAGGAAGAACACAATGACGGTACTTGAGGAATAAGCCTCGGCTAACTCCGTGCCAGCAGCCGCGGTAATACGGAGGAGGCAAGCGTTATCCGGAATGATTGGGCGTAAAGCGTCCGTAGGTGGCAGTTCAAGTCTGCTGTCAAAGACAGTAGCTCAACTACTGAAAGGCAGTGGAAACTGAACAGCTAGAGTACGGTAGGGGCAGAGGGAATTCCCGGTGTAGCGGTGAAATGCGTAGATATCGGGAAGAACACCGGTGGCGAAAGCGCTCTGCTGGGCCGTAACTGACACTGAGGGACGAAAGCTAGGGGAGCGAATGGGATTAGATACCCCAGTAGTCCTAGCCGTAAACGATGGAAACTAGGTGTAGCCTGTATCGACCCGGGCTGTGCCGAAGCTAACGCGTTAAGTTTCCCGCCTGGGGAGTACGCACGCAAGTGTGAAACTCAAAGGAATTGACGGGGGCCCGCACAAGCGGTGGAGTATGTGGTTTAATTCGATGCAACGCGAAGAACCTTACCAGGGCTTGACATGTCCGGAATCTTGGTGAAAGCCGAGAGTGCCTTCGGGAGCCGGAACACAGGTGGTGCATGGCTGTCGTCAGCTCGTGTCGTGAGGTGTTGGGTTAAGTCCCGCAACGAGCGCAACCCTCGTCCTTAGTTGCCATCATTCAGTTGGGCACTTTAGGGAGACTGCCGGTGACAAACCGGAGGAAGGTGGGGATGACGTCAAGTCATCATGCCCCTTACGTCCTGGGCTACACACGTACTACAATGGGGGGGACAAAGGGTAGCCAAGACGCGAGTCTGAGCCAATCCCGTAAACCTCTCCTCAGTTCAGATTGCAGGCTGCAACTCGCCTGCATGAAGGAGGAATCGCTAGTAATCGCAGGTCAGCATACTGCGGTGAATCCGTTCCCGGGCCTTGTACACACCGCCCGTCACACCATGGAAGTTAGCCACGCCCGAAGTCGTTACTCTAACCGTTCGCGGAGGAGGATGCCGAAGGCAGGGCTGATGACTGGGGTGAAGTCGTAACAAGGTAGCCGTACCGGAAGGTGTGGCTGGATCACCTCCTTTTTAGGGAGACCTACTTCGAGATATCGCGCCTTAACAACTATAGCCGTGTCTTGAGGTCATCCTTAGGTCGGATGGGGCGGTCAGAGAGCTTTCAAACTTTAGGGTTCGTGTTATGGGCTATTAGCTCAGGTGGTTAGAGCGCACCCCTGATAAGGGTGAGGTCCCTGGTTCAAGTCCAGGATGGCCCACATCCACCCCAAACTGGGGGTATAGCTCAGTTGGTAGAGCGCTGCCTTTGCACGGCAGAAGTCAGCGGTTCGAGTCCGCTTACCTCCACTCTCCTTTGTGATGGTGCTAGTTGGGGTGAGATGAGATGAGATGACCTCTGATAGATAATTTATCACTGTACAGCTCCTAAATCTTTAGATGTTAGTCTGAGATTGGATAGCTGGACATCTGTTCCAGTCAGAACCTTGAAAACTGCATAGAGAAAAGCATAATGGTGTAGGAAAACGTCGTAAAGACAATTCCAATGTAGGTCAAGCTACAAAGGGCTAACGGTGGAACCTAGGCACACAGAGCGGCCGCAAA

## Appendix C. Sequences of the 18S Ribosomal RNA Gene of the Studied Microorganism (Sample III)

CTGGTTGATCCTGCCAGTAGTCATATGCTTGTCTCAAAGATTAAGCCATGCATGTCTAAGTATAAACTGCTTATACTGTGAAACTGCGAATGGCTCATTAAATCAGTTATAGTTTATTTGATGGTACCTTTACTCGGATAACCGTAGTAATTCTAGAGCTAATACGTGCGTAAATCCAGACTTCTGGAAGGGACGTATTTATTAGATAAAAGGCCAGCCGGGCTTGCCCGACTCTTGGCGAATCATGATAACTTCACGAATCGCACGGCTTTATGCCGGCGATGTTTCATTCAAATTTCTGCCCTATCAACTTTCGATGGTAGGATAGAGGCCTACCATGGTGGTAACGGGTGACGGAGGATTAGGGTTCGATTCCGGAGAGGGAGCCTGAGAAACGGCTACCACATCCAAGGAAGGCAGCAGGCGCGCAAATTACCCAATCCCAACACGGGGAGGTAGTGACAATAAATAACAATACCGGGCATTTTTGTCTGGTAATTGGAATGAGTACAATCTAAATCCCTTAACGAGTATCCATTGGAGGGCAAGTCTGGTGCCAGCAGCCGCGGTAATTCCAGCTCCAATAGCGTATATTTAAGTTGTTGCAGTTAAAAAGCTCGTAGTTGGATTTCGGGTGGGTTGTAGCGGTCAGCCTTTGGTTAGTACTGCTACGGCCTACCTTTCTGCCGGGGACGAGCTCCTGGGCTTAACTGTCCGGGACTCGGAATCGGCGAGGTTACTTTGAGTAAATTAGAGTGTTCAAAGCAAGCCTACGCTCTGAATACATTAGCATGGAATAACACGATAGGACTCTGGCTTATCTTGTTGGTCTGTAAGACCGGAGTAATGATTAAGAGGGACAGTCGGGGGCATTCGTATTTCATTGTCAGAGGTGAAATTCTTGGATTTATGAAAGACGAACTTCTGCGAAAGCATTTGCCAAGGATGTTTTCATTAATCAAGAACGAAAGTTGGGGGCTCGAAGACGATTAGATACCGTCGTAGTCTCAACCATAAACGATGCCGACTAGGGATTGCCAGGTGTTTCGTTGATGACCCTGCCAGCACCTTATGAGAAATCAAAGTTTTTGGGTTCCGGGGGGAGTATGGTCGCAAGGCTGAAACTTAAAGGAATTGACGGAAGGGCACCACCAGGCGTTAACTTAGCAGCAAGCTCAGCGCCTCAAAGTCGAAGGGAAACCTTTGGCTAGTATCTGGGTGTAGATTTCACCTAAGTGCAACACTGTTCAAATTGCGGGAAAGCCCTAAAGCTTTGCTAACCAAGCTGTCCTAGAAATGGGATGGTGGCCAGGTGAAAGACCTTGGGTACGGTAAAATCAGCAAAGATGCAACAATGGGCAATCCGCAGCCAAGCTCCTACGGGCTGTCAAAGCCTATGGAGAAGGTTCAGAGACTAAATGGCAGTGGGCAAGCATGGCAATGCTTGCTTAAGATATAGTCCGTCCCAGCTGAGAAGCTGCCTATGAGAGGAATGCCGTAAGGCAGGAGAGCTAATAGGAAGTAAGTGTCTTTAATCAACTTACTTGGATTCCACGGGAGCCTGCGGCTTAATTTGACTCAACACGGGAAAACTTACCAGGTCCAGACACGGGGAGGATTGACAGATTGAGAGCTCTTTCTTGATTCTGTGGGTGGTGGTGCATGGCCGTTCTTAGTTGGTGGGTTGCCTTGTCAGGTTGATTCCGGTAACGAACGAGACCTCAGCCTGCTAAATAGTCACGTCTACCTCGGTAGGCGCCTGACTTCTTAGAGGGACTATTGGCGTTTAGCCAATGGAAGTGTGAGGCAATAACAGGTCTGTGATGCCCTTAGATGTTCTGGGCCGCACGCGCGCTACACTGATGCATTCAACGAGCCTATCCTTGGCCGAGAGGTCCGGGTAATCTTTGAAACTGCATCGTGATGGGGATAGATTATTGCAATTATTAGTCTTCAACGAGGAATGCCTAGTAAGCGCGAGTCATCAGCTCGCGTTGATTACGTCCCTGCCCTTTGTACACACCGCCCGTCGCTCCTACCGATTGGGTGTGCTGGTGAAGTGTTTGGATCGGTACCAATGGGGGGAAACCTCTGTTGGTACTGAGAAGAACATTAAACCCTCCCACCTAGAGGAAGGAGAAGTCGTAACAAGGTTTCCGTAGGTGAACCTGCAGAAGGATCA
